# The Role of Visual Eccentricity on Preference for Abstract Symmetry

**DOI:** 10.1371/journal.pone.0154428

**Published:** 2016-04-28

**Authors:** Giulia Rampone, Noreen O’ Sullivan, Marco Bertamini

**Affiliations:** 1 University of Liverpool, Department of Psychological Sciences, Eleanor Rathbone Building, University of Liverpool, Liverpool, L69 7ZA, United Kingdom; 2 Liverpool John Moores University, Institute of Natural Sciences & Psychology, School of Natural Sciences & Psychology, Byrom Street, Liverpool, L3 3AF, United Kingdom; University of Pécs Medical School, HUNGARY

## Abstract

This study tested preference for abstract patterns, comparing random patterns to a two-fold bilateral symmetry. Stimuli were presented at random locations in the periphery. Preference for bilateral symmetry has been extensively studied in central vision, but evaluation at different locations had not been systematically investigated. Patterns were presented for 200 ms within a large circular region. On each trial participant changed fixation and were instructed to select any location. Eccentricity values were calculated a posteriori as the distance between ocular coordinates at pattern onset and coordinates for the centre of the pattern. Experiment 1 consisted of two Tasks. In Task 1, participants detected pattern regularity as fast as possible. In Task 2 they evaluated their liking for the pattern on a Likert-scale. Results from Task 1 revealed that with our parameters eccentricity did not affect symmetry detection. However, in Task 2, eccentricity predicted more negative evaluation of symmetry, but not random patterns. In Experiment 2 participants were either presented with symmetry or random patterns. Regularity was task-irrelevant in this task. Participants discriminated the proportion of black/white dots within the pattern and then evaluated their liking for the pattern. Even when only one type of regularity was presented and regularity was task-irrelevant, preference evaluation for symmetry decreased with increasing eccentricity, whereas eccentricity did not affect the evaluation of random patterns. We conclude that symmetry appreciation is higher for foveal presentation in a way not fully accounted for by sensitivity.

## Introduction

Bilateral symmetry is a ubiquitous structural property of objects, which is salient both for humans and for other animal species [1–5]. It has been suggested that the visual system is particularly tuned to bilateral symmetry and uses this property as a perceptual cue in figure-ground discrimination (e.g. [6–8]). Bilateral symmetry in clouds of dots is accurately distinguished from random dot patterns of similar size and density at brief exposure times [9–11], even when embedded in noise [11–13]. As bilateral symmetry is *effortlessly* extracted [14], it has been suggested that it acts as a visual primitive and it has been incorporated as a Gestalt property [15,16].

The association between symmetry (and in particular bilateral symmetry) with beauty is longstanding. Many animal species, including humans, use symmetry as a biological signal of mate quality [5,17–21]. Ramachandran and Hirstein [22] proposed symmetry as a basic principle of aesthetics and artistic experience. Symmetry is a good predictor of preference when people evaluate the aesthetic appeal of abstract patterns [23,24]. Moreover, there is evidence of automatic association between symmetry and positive valence [25–28].

This strong, and possibly innate [29], aesthetic appreciation of symmetry may derive from the ease of its processing (*perceptual fluency hypothesis*, [30]).

Bilateral symmetry is the optimal stimulus to activate a regularity-specific extrastriate visual network, although it is unlikely that a symmetry-specific area exists [31]. Moreover, there is no evidence that symmetry is extracted by low level-visual mechanisms (like V1 and V2 [32–35]). When the regularity around the axis of symmetry is less accessible, the saliency of symmetry drastically reduces. For example, symmetry detection is possible in extra-foveal vision but with reduced sensitivity [9,11,36,37]. Symmetry detection declines rapidly as a function of increasing retinal eccentricity (although appropriate size scaling removes the eccentricity dependence) [12,38–40].

If increasing eccentricity gradually impairs detection of symmetry, it would seem plausible that aesthetic appreciation of symmetry decreases with a similar trend. Because foveal perception is important for fluently extracting the information around the bilateral axis of symmetry and constructing the representation of shape, people would need to look at bilateral symmetry foveally to appreciate it. However, to the best of our knowledge there has been no systematic study of the change in aesthetic appreciation of bilateral symmetry across retinal eccentricities.

It is generally accepted that beauty can be easily detected in extrafoveal vision (e.g. Kuraguchi and Ashida [41] and Guo and coll. [42] conducted studies on detection of beautiful faces in the periphery), and beauty in the periphery captures attention even when it is task irrelevant [43]. It is possible therefore that symmetry is detected and preferred to non-symmetry in the periphery (as long as it can be discriminated). The aim of this study is to compare the affective value that people attribute to the same type of regularity presented at different levels of eccentricity. Moreover, we wanted to test the link between the decrement in preference with increasing eccentricity and the difficulty in processing symmetry (measured by manual reaction times and proportion of incorrect responses).

We used abstract patterns made of black dots. These could have either bilateral symmetry (with both vertical and horizontal axes of reflection) or random configuration. On each trial, one pattern (4.6° of visual angle) appeared for 200 ms at a random location within a large circular region (25.6°). In order to reduce the artificiality of the experiment, participants autonomously chose a fixation point (not marked) within the circle prior to pattern onset. The participant changed fixation at the beginning of each trial. Hence, the distance from the centre of the pattern and the fovea was not controlled by the experimenter and could not be predicted by the participant. The value of retinal eccentricity was calculated a posteriori and varied across trials and across participants. However, for all participants, the final eccentricity values ranged approximately between 0 and 18 degrees of visual angle. Experiment 1 was divided in two tasks. In Task 1 participants classified the regularity of the patterns (as “random” or “symmetry”) immediately after pattern offset. In the second task (Task 2) participant did not classify pattern’s regularity but rated the pattern on a 9-point liking scale after pattern offset. Note that patterns presented in Task 1 were different from those presented in Task 2. In this way we eliminated any bias due to familiarity (*mere exposure effect* [44])

In Task 1 manual reaction times and response errors were recorded. These were used as measures of the perceptual impairment caused by retinal eccentricity. We expected an increase in response errors and latency with increasing eccentricity. In addition, Task 1 allowed participants to familiarize to the type of patterns and reduce the effort required to discriminate regularity in Task 2. We were aware that in this experiment patterns at larger eccentricities might be misclassified (e.g. symmetry could be confounded with random, or vice versa) and misevaluated accordingly. The practice in Task 1 helped to maximise correct discrimination of regularity at the farthest eccentricities.

We can distinguish three possible outcomes.

(1) Eccentricity may fail to predict evaluation. Participants might rate symmetry more positively (ratings from 5 to 9) than random (ratings form 5 to 1) at any eccentricity. This category-based evaluation would suggest that regularity is the sole predictor of preference modulation, whereas the reduced saliency caused by eccentricity does not influence preference.

(2) Eccentricity may cause a decrease in rating only for symmetry, but not for random patterns. This would suggest that the aesthetic appreciation of symmetry benefits from foveal processing.

(3) Finally, another possible outcome would be that eccentricity generally predicts more negative evaluations for all stimuli. This would imply a general preference for central presentations.

Experiment 2 was conducted to test the effect of eccentricity on the evaluation of symmetry (and random) presented in isolation and not confronted with its counterpart. The experimental design was similar to Experiment 1. One group of participants observed only symmetric patterns and the other group observed random patterns. Patterns were made of black and white dots and participants reported whether the pattern contained more black or more white dots. Immediately after a response, participants evaluated their liking for the pattern on a 9-points rating scale. If retinal eccentricity is a predictor of liking for regular patterns but not for random patterns, a linear relationship between ratings and eccentricity will be observed only in the group that saw symmetric patterns. Another advantage of this task was to measure evaluation of symmetry across eccentricity when symmetry was task-irrelevant.

To summarise, this study aimed to answer the following questions: (Q1). Is eccentricity a general predictor of lower preference or is it specifically detrimental for the aesthetic appreciation of regular patterns (bilateral symmetry)? (Q2). Does eccentricity affect evaluation by impairing the discrimination of symmetry at peripheral locations?

## Experiment 1

To investigate the effect of eccentricity on the appreciation of symmetry, participants were presented with abstract patterns made of black dots, with either two-fold bilateral symmetry or a random configuration. On each trial one pattern was presented for 200 ms inside a large grey circle in the centre of the screen. The coordinates for the position of the pattern were randomly generated on each trial. Participants were asked to change fixation on each trial arbitrarily. Eccentricity values were calculated a posteriori, and a different array of eccentricity values was obtained from each participant. This method was employed to reduce the artificiality of the experimental design. Task 1 tested manual response speed and accuracy in symmetry detection as a function of retinal eccentricity. The same participants performed Task 2. In this task participants did not perform a classification task. They evaluated their liking for each pattern on a 9-points Likert rating scale. This second part tested whether increasing eccentricity predicted a reduction in preference for patterns.

In order to avoid familiarity influences on aesthetic evaluation, we ensured that patterns were generated afresh on each trial and they differed in Task 1 and Task 2. Therefore participant never saw the same pattern twice. Eccentricity was calculated as the distance from eyes coordinates at pattern onset and the coordinates of the centre of the pattern inside the circle.

### Method

#### Participants

Twenty participants from the cohort of psychology students at the University of Liverpool participated in both experiments (age 18–31 years, mean age 19 years, 1 male, 2 left handed). All were naïve in respect to the experimental hypotheses and had normal or corrected to normal vision. They provided written consent for taking part and received course credits. The experiment was approved by the Ethics Committee of the University of Liverpool and was conducted in accordance with the Declaration of Helsinki (2008).

#### Apparatus and stimuli

Participants sat at 57 cm from a 16-inch LCD monitor with resolution 1280 X 1024 pixels at 75 Hz. To prevent loss of data due to head movements, a chin rest was employed to keep the head still. Participants’ eye movements were measured using an ASL Eye-Trac D6 (Applied Science Laboratories, Bedford, MA) at a sampling rate of 120 Hz.

**Fig 1:**
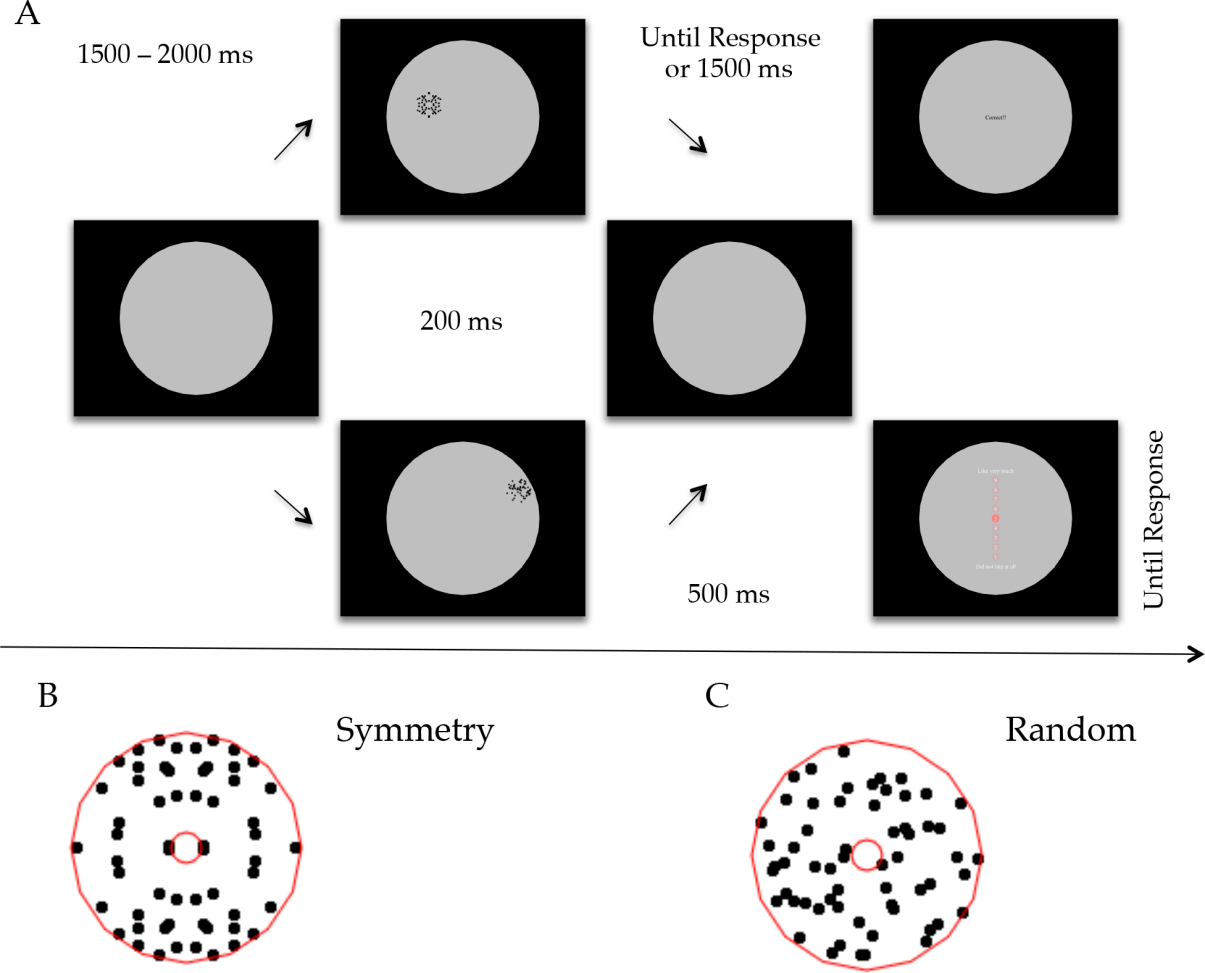
(**A**) Experimental procedure of Experiment 1. Each trial started with an interval between 1500 ms and 2000 ms. During this interval the participant could look at any location within the central large circle, and chose a point where maintaining the gaze. After the interval an abstract pattern appeared at a random location. The pattern could be either symmetry or random, and remained on the screen for 200 ms. Participants were encouraged to control the reflexive response to look at the pattern and maintain fixation on the point they chose. Task 1: Immediately after pattern offset, participants reported whether the pattern was symmetry or random. If no response was given within 1500 ms from pattern offset, the trial was considered null and a new trial started. Task 2: After 500 ms from pattern offset, a 9-points rating scale was presented. Participants moved the cursor up and down on the rating scale to assign a preference value to the pattern (9 = like very much; 1 = do not like a t all). They were encouraged to give a response relatively fast and using a gut feeling. (**B**) Example of a symmetry pattern. (**C**) Example of a random pattern. Red lines indicate the virtual circles used to construct the patterns. These were not visible to the participants and are shown here for illustrative proposes.

Stimuli were generated using PsychoPy software [45] and consisted of abstract black-dots patterns with either symmetrical or random configuration. Each pattern was composed of 60 dots arranged within a region delimited by two virtual circular perimeters (as indicated by the red lines within the pattern in Fig 1B and 1C). The radius of the internal small circle was 0.2° of visual angle; the radius of the external circle was ~1.5°. Therefore the global size of the patterns was approximately ~3°. Each dot had radius 0.1°. Symmetric patterns were constructed by randomizing the arrangement of the dots in one of four quadrants. Each quadrant contained 15 dots. In this way we obtained bilateral symmetry both on the vertical and horizontal axis. We choose to use a two-fold bilateral symmetry to maximize the saliency of the symmetrical patterns. This was intended to reduce the possibility of misclassification between symmetry and random at greater eccentricities, which could bias evaluation (Fig 1B). For random patterns the arrangement of the dots inside each quadrant was unconstrained (Fig 1C).

Stimuli were presented within a grey circle (RGB 0,0,0) with radius 12.8°. Coordinates of stimulus position were randomly generated on each trial. The pattern could appear at any position within the circle. Because pattern position changed on each trial, it could not be predicted. Participants used a gamepad to report their response accordingly to task instructions.

The experiment was divided in two tasks. In Task 1 participants pressed the two top-bottom shoulder buttons of the gamepad (7–8) to report pattern regularity (symmetry or random). In Task 2 a Likert vertical scale was presented after stimulus appearance at the centre of the screen. It consisted of a column of numbers from 9 to 1, headed by the messages “Like it very much” at the top, and “did not like at all” at the bottom. Participants could move up and down on the scale using the arrows on the gamepad. The position on the scale was indicated by a change in opacity of the circle surrounding the specific number. Participants confirmed their final response by pressing button 1 on the gamepad.

#### Procedure

Fig 1A illustrates the experimental procedure. A large grey circle over a black background delimited the area of interest. Each trial started with a variable interval of 1500 to 2000 ms. During this interval participants were required to choose arbitrarily any point within the grey circle and keep fixation on that point. An abstract pattern appeared at an unpredicted position within the grey circle. Participants were instructed to try to control reflexive saccadic responses to pattern onset. In Task 1, participants classified the pattern as symmetry or random as fast and accurately as possible. One group of participants pressed a left button for symmetry and right button for random. The other group did the opposite. Response screen was displayed until response. A feedback word (“correct” or”incorrect”) was displayed for 500 ms immediately after response, then a new trial started. Task 2 was identical except that participants did not classify pattern regularity and maintained fixation until a vertical 9-points rating scale was presented. Participants were encouraged to base their evaluation on a first spontaneous reaction to the pattern.

Each task consisted of 144 trials, divided in four blocks of 36 trials. Between each block participants were allowed to rest and disengage the eyes from the screen. The two tasks followed one after the other with a break (~5/10 min) between them and always with the same order. The order of the two tasks was not counter-balanced. Task 1 always preceded Task 2. This was intentional in order to facilitate sensitization to symmetrical stimuli for the preference task. A practice session of 20 trials preceded Task 1, whereas a practice session of 10 trials preceded Task 2. These reproduced the procedure of the incoming Task, in order to ensure participants understood the instructions. A questionnaire was provided at the end of both experiments asking participants their personal opinion about the purpose of the study. This was used to ensure participants did not understand the real experimental aims.

#### Analysis

Spatial coordinates of stimulus position were calculated as the distance between the ocular coordinates at target onset and the coordinates of the center of the circular region in which patterns were embedded. Eccentricity values ranged approximately between 0 and 19 degrees of visual angle (Task 1 M = 8.84; SD = 3.8; range = 17.99; Task 2 M = 8.88; SD = 3.9; range = 19.121). We discarded trials in which eyes’ signal was not recorded (Task 1 5.6%, Task 2 10%, of total trials), or incorrect eye movements were performed during pattern presentation (Task 1 4.4%, Task 2 6%, of total trials). In Task 1, 10% of total trials were excluded from the analysis, In Task 2 we excluded 16% of total trials. However, the average proportion of excluded trials in the symmetry and random conditions did not differ in both experiments.

Multi-level linear modelling is a statistical approach for hierarchical data sets in which data is sampled at different levels of a hierarchy. In our study experimental trials (the lowest level of the hierarchy) were nested within participants (the highest level of the hierarchy). In contrast to a standard regression model in which the dependent variable is a measure of central tendency that is detached from the variance around that score, a multi-level model includes estimates of the variance at each level of a hierarchical data-set, adjusting the estimates of other parameters in the model accordingly. Random effects in the model relate to the extent that variance in the dependent variable (DV) can be attributed to variance at a particular level in the hierarchy (e.g. a random effect of participant; a random effect of trial). Fixed effects in the model relate to the extent that variance in the DV can be attributed to a manipulated variable. Through partitioning the variance in this way within the context of one model, the parameters that are estimated for the fixed effects are statistically unbiased by, for example, variability across participants.

Fixed factors in our analysis were eccentricity, pattern regularity, and two parameters that we called Mean Individual Eccentricity (MIE) and Mean Individual Inverse Efficiency Score (MIIES). MIE corresponds to the mean distance from eyes at which patterns were presented, calculated for each participant. As eccentricity values were not pre-set and balanced, each participant might have been exposed to patterns at different eccentricity. Taking this variable into account as fixed variable of our model, allowed us to control for individual differences in eccentricity exposure.

MIIES corresponds to the mean “inverse efficiency score” [46,47] for each participant. MIIES integrates the average proportion of correct responses (PC) and latency of correct responses (reaction times, RTs) in a unique variable, in order to weight the impact of speed and accuracy. For each participant mean RT was divided by mean PC, the value obtained corresponds to MIIES and it is expressed in ms (like RT). This variable was included in the model employed for the analysis of preference evaluation. Including MIIES allowed controlling for the effect of individual efficiency in performing Task1 on preference evaluation.

Two multilevel linear models were employed to analyse reaction times (Task 1) and preference ratings (Task 2). Accuracy (Task 1) is a binary dependent variable; therefore a binary logistic model was performed. Correct responses were coded as 1, and incorrect responses as 0. The model for RTs included only trials in which a correct response was made. Each DV was analysed as a function of increasing eccentricity and pattern regularity. All models revealed that random factors (participants and trials numbers) generated significant variability in the data (all ps <0.05). However, we were not interested in testing the role of random factors on the final outcome. Therefore these will not be discussed any further. The sampling distribution of the t-statistic is the Student’s t-distribution. Therefore, degrees of freedom were calculated as *n-k*, where *n* is sample size and *k* is the number of parameters in the model. The analysis was carried out in MLwiN [48].

### Results

#### Task 1

**Fig 2:**
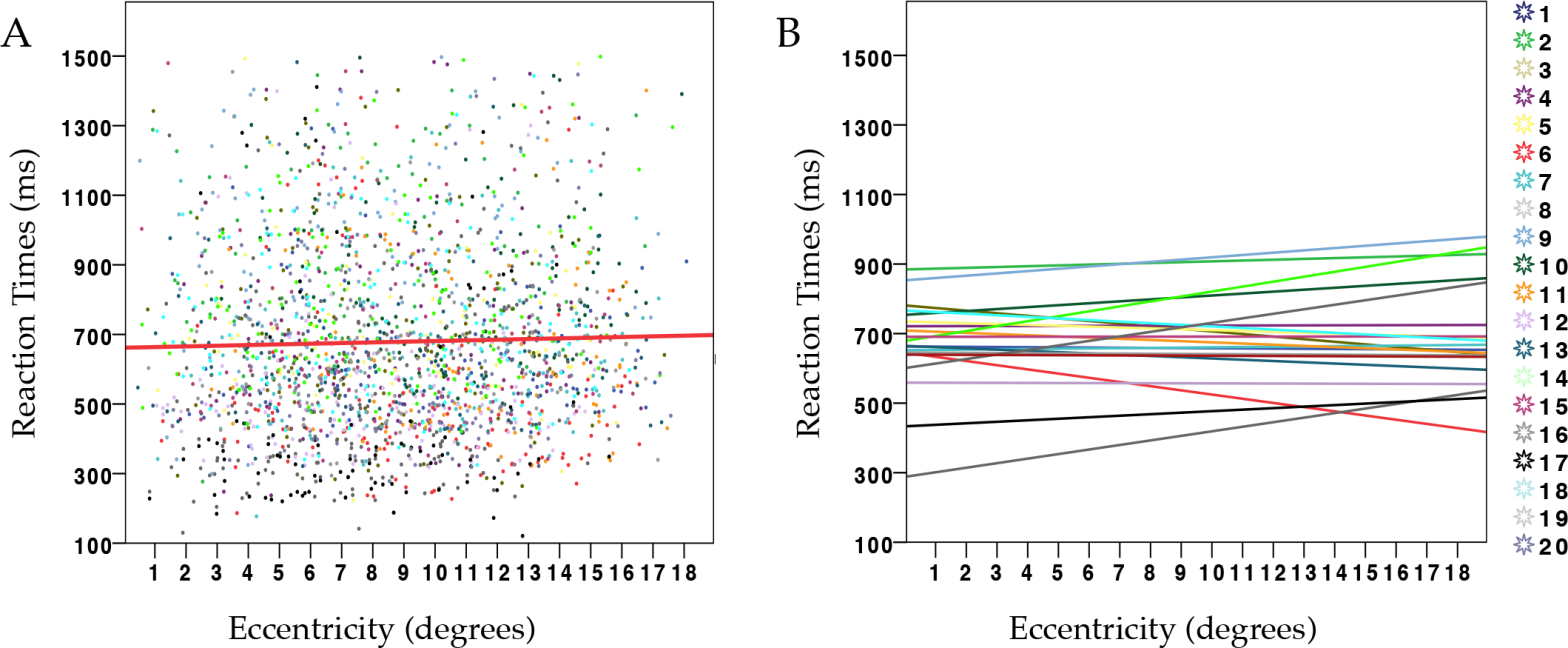
(**A**) Scatterplot showing the relationship between manual reaction times (RTs) and retinal eccentricity across all trials and participants, with regression line (red) corresponding to the average relationship (R2 = 0.0008). (**B**) Each line corresponds to the relationship between RTs and eccentricity *within* each participant.

Table 1 shows results from the model used for analysis of RTs. Overall both eccentricity and pattern regularity were unrelated to variability in reaction times (see Table 1 and Fig 2).

**Table 1:** Results from analysis of reaction times.

	β	SE	t	p
**Intercept**	0.7	0.03	23.9	<0.001
**MIE**	0.03	0.03	0.9	0.4
**Eccentricity**	0.002	0.002	1.0	0.3
**Pattern regularity (symmetry)**	0.02	0.01	1.7	0.1
**Eccentricity x Pattern regularity**	-0.002	0.003	-0.7	0.5

Results from Multilevel Linear Model for analysis of reaction times as a function of visual eccentricity in Experiment 1 (Task 1).

Correct responses were the 85.5%. Table 2 shows results from the Binary Logistic Model for the analysis of correct responses as function of eccentricity. Increasing eccentricity did not affect the odds of correct responses (OR = .96, *Χsq* (1, *N* = 20) = 3.7, *p* = 0.06). Instead, the odds of correct responses significantly decreased when symmetry was presented (OR = .78, *Χsq* (1, *N* = 20) = 5.0, *p* = 0.03), suggesting a possible bias in classifying the patterns as random. However, the interaction Eccentricity X Pattern Regularity was not significant (OR = .98, *Χsq* (1, *N* = 20) = 0.5, *p* = 0.5).

**Table 2:** Results from analysis of correct responses.

	β	SE	Odd Ratio	prob	*Χsq*	p
**Intercept**	1.9	0.15				
**MIE**	0.3	0.2	1.35	0.95	3.2	0.085
**Eccentricity**	-0.04	0.02	0.96	0.87	3.7	0.06
**Pattern regularity (symmetry)**	-0.25	0.12	0.78	0.85	5.0	0.03
**Eccentricity x Pattern regularity**	-0.02	0.03	0.98	0.87	0.5	0.5

Results from Binary Logistic Model for analysis of correct responses as a function of visual eccentricity in Experiment 1 (Task 1).

#### Task 2

**Fig 3:**
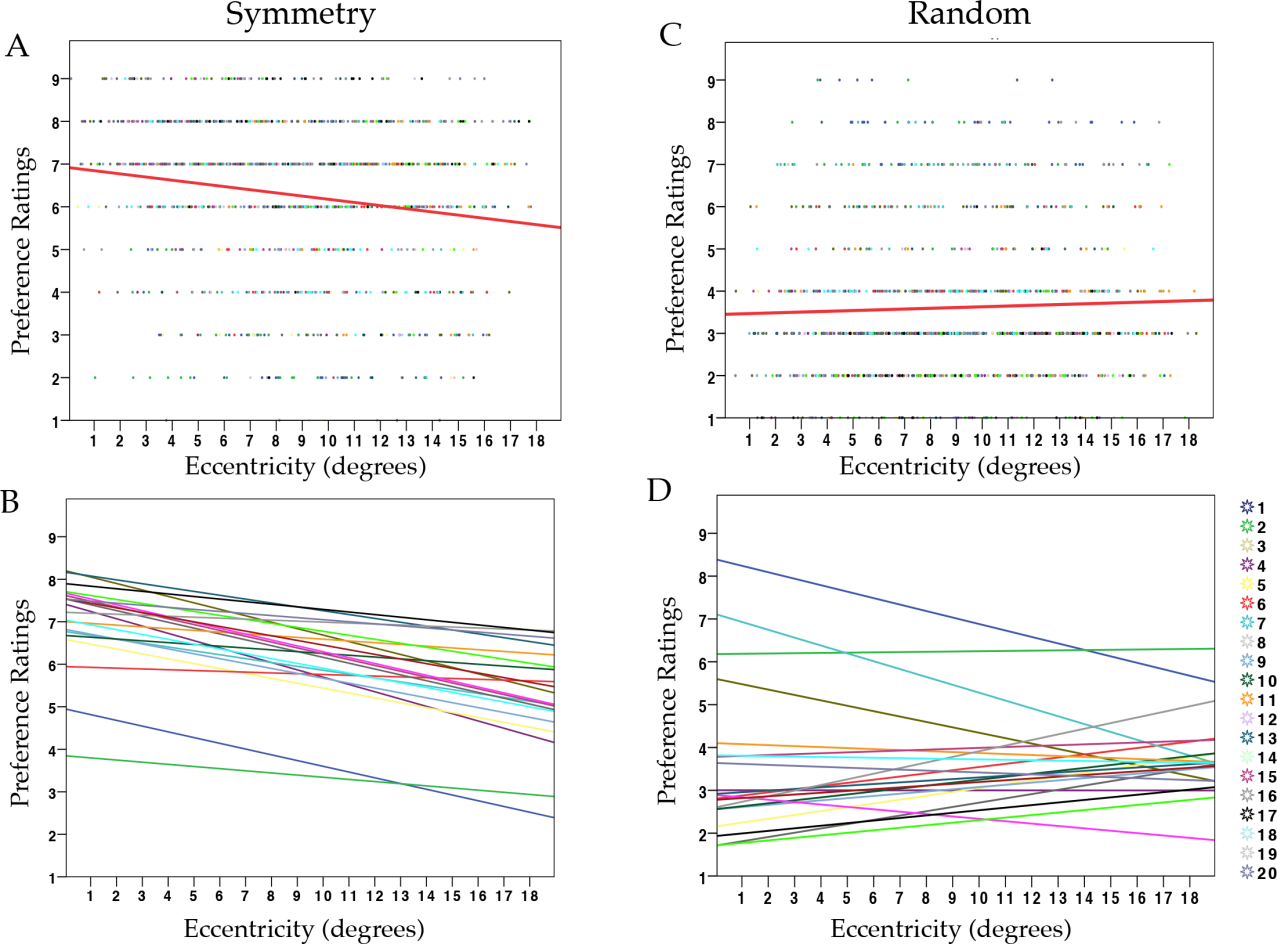
Results on preference evaluation in Experiment 1, Task 2. (**A**) Scatterdot plot showing preference ratings (1–9) for symmetry in relation to retinal eccentricity across all trials and participants, with regression line (red) (R2 = 0.03). **(B)** Each line corresponds to the relationship between preference and eccentricity within each participant for symmetry patterns **(C)** Scatterdot plot showing preference ratings (1–9) for random in relation to retinal eccentricity, across all trials and participants, with regression line (red) (R2 = 0.002). **(D)** Each line corresponds to the relationship between preference and eccentricity within each participant for random patterns.

We ran one model using liking ratings as the dependent variable. This model included one more fixed factor: MIIES (see **Analysis** session). Results are shown in Table 3. The model suggests that Pattern Regularity was a good predictor for preference evaluation (t_(14)_ = 37.4, p <0.001). Increasing eccentricity was not a predictor of preference formation overall (t_(14)_ = 0.08, p .94). However, there was an interaction Eccentricity * Pattern Regularity (t_(14)_ = -4.94, p <0.001). Table 4 shows results from two separate models for Random and Symmetry conditions. Preference for symmetry decreased with increasing eccentricity (t_(17)_ = -7.7, p <0.001), whilst the same was not true for random stimuli (t_(17)_ = 0.8, p = 0.4). See Scatterplots in Fig 3A and 3B (symmetry) and Fig 3C and 3D (random).

**Table 3:** Results from analysis of preference ratings.

	β	SE	t	p
**Intercept**	3.6	0.09	39.3	<0.001
**MIE**	0.2	0.07	2.2	0.05
**MIIES**	<0.001	0.001	<0.001	1
**Eccentricity**	0.001	0.01	0.08	0.9
**Pattern regularity**	2.65	0.07	37.4	<0.001
**Eccentricity x Pattern regularity**	-0.09	0.02	-4.9	<0.001

Results from Multilevel Linear Model for analysis of preference ratings as a function of visual eccentricity in Experiment 1 (Task 2).

**Table 4:** Analysis of preference ratings separately for Random and Symmetry patterns.

	Random				Symmetry			
** **	**β**	**SE**	**t**	**p**	**β**	**SE**	**t**	**p**
**Intercept**	3.7	0.3	13.5	<0.001	6.2	0.2	28.5	<0.001
**MIE**	0.08	0.2	0.4	0.7	0.2	0.1	1.3	0.2
**Eccentricity**	0.008	0.01	0.8	0.4	-0.09	0.01	-7.7	<0.001

Results from Multilevel Linear Model for analysis of preference ratings as a function of visual eccentricity separately for Random and Symmetry patterns (Experiment 1 (Task 2)).

MIE significantly affected preference. This suggests that participants that were more often exposed to patterns at larger eccentricities tended to use higher ratings overall.

### Discussion

Results from Task 1 revealed that eccentricity did not affect either accuracy or reaction times. We did not observe any effect of visual eccentricity on detection speed and accuracy for symmetry against random patterns. This is not in agreement with previous findings [36,38,49], but it is possible that the type of design was not ideal for recording manual response speed. We will discuss this aspect in the General Discussion. Results from Task 2 showed that Eccentricity did not predict lower ratings in general. A significant Eccentricity X Pattern Regularity interaction showed, instead, that eccentricity differently modulated the evaluation of the two types of regularity. Ratings for symmetry decreased to more negative values with increasing eccentricity, whereas ratings for random patterns remained unvaried. This supports the hypothesis that proximity to the fovea is important for the aesthetic appreciation of bilateral symmetry.

However, an important caveat in this result is that evaluation may have been subject to a regression to the mean. Because symmetric and random patterns were interleaved, it is possible that patterns at farther distances were more often misclassified and rated accordingly (i.e. symmetry was confounded and rated as random and vice versa). There was no significant evidence of a regression to the mean in the ratings for random patterns (i.e. evaluation for random patterns did not gradually become more positive with eccentricity). However, as shown in Fig 2B and 2D, individual regression lines for both categories suggests (descriptively) a weak tendency toward converging.

We also observed that in Task 1 participants were significantly more prone to classify patterns as random in case of doubt. However, and relevant for the current study, pattern regularity did not interact with eccentricity. This means that increasing eccentricity did not cause the reduction in the odds of correct responses to symmetry, and this bias was generalized at any eccentricity level. In the method session we explain that two-fold reflection symmetry was used with intention of maximising the saliency of regularity. The fact that participants often perceived symmetric patterns as random was thus unexpected. Potentially, other factors may have influenced this bias (e.g. the absence of a fixation mark; a high degree of spatial unpredictability, due to randomised patterns’ location combined with arbitrary choice of fixation point). However, no specific prediction was made about the role played by any of these factors on detection accuracy, and this hypothesis requires proper investigation.

The fact that symmetry was significantly liked more than random confirms that the different regularities were correctly discriminated in the evaluation task. At this stage, therefore, it was important to clarify whether misperception of symmetry at farther eccentricities caused the interaction between Eccentricity and Pattern Regularity on preference ratings. Experiment 2 was similar to Experiment 1. This time one group of participants saw only symmetric patterns, whereas the other group saw only random patterns. In this way any confound due to misclassification of Pattern Regularity was avoided.

## Experiment 2

Experiment 1 suggested that increasing eccentricity leads to devaluation of symmetry, but not random patterns. However, it is possible that symmetry at farther retinal locations was more often confounded and evaluated as random. The second experiment was conducted to investigate this possibility. We introduced four important changes. First, one group of participants saw only symmetry (Experiment 2a), whereas the other group saw only random (Experiment 2b). Second, patterns were made of a random number of black and white dots. Participants reported whether the pattern contained more black dots or more white dots. Third, the evaluation task was included in the same experiment. Immediately after responding to the proportion of dots with same luminance polarity, participants rated how much they liked the patterns that they had just seen. Four, the starting point of the rating scale was moved from 5 (central value on the rating scale) to 1 (lowest value of the rating scale). In this way we assumed that evaluation was made following a criterion of ‘gradual distance from the starting point’. Note that the detection task served to disguise participants from the real aim of the experiment. No predictions were made regarding the evaluation of different proportions of black/white dots.

Forty people took part in Experiment 2 (aged 18 to 22, 6 males, 4 left handed). They were all first year students from the School of Psychology of the University of Liverpool, and received course credits. They provided written consent. The experiment received ethics approval and was conducted in accordance with the Declaration of Helsinki (2008). Twenty participants took part in Experiment 2a, the other participants did Experiment 2b.

### Experiment 2a

#### Method

**Fig 4:**
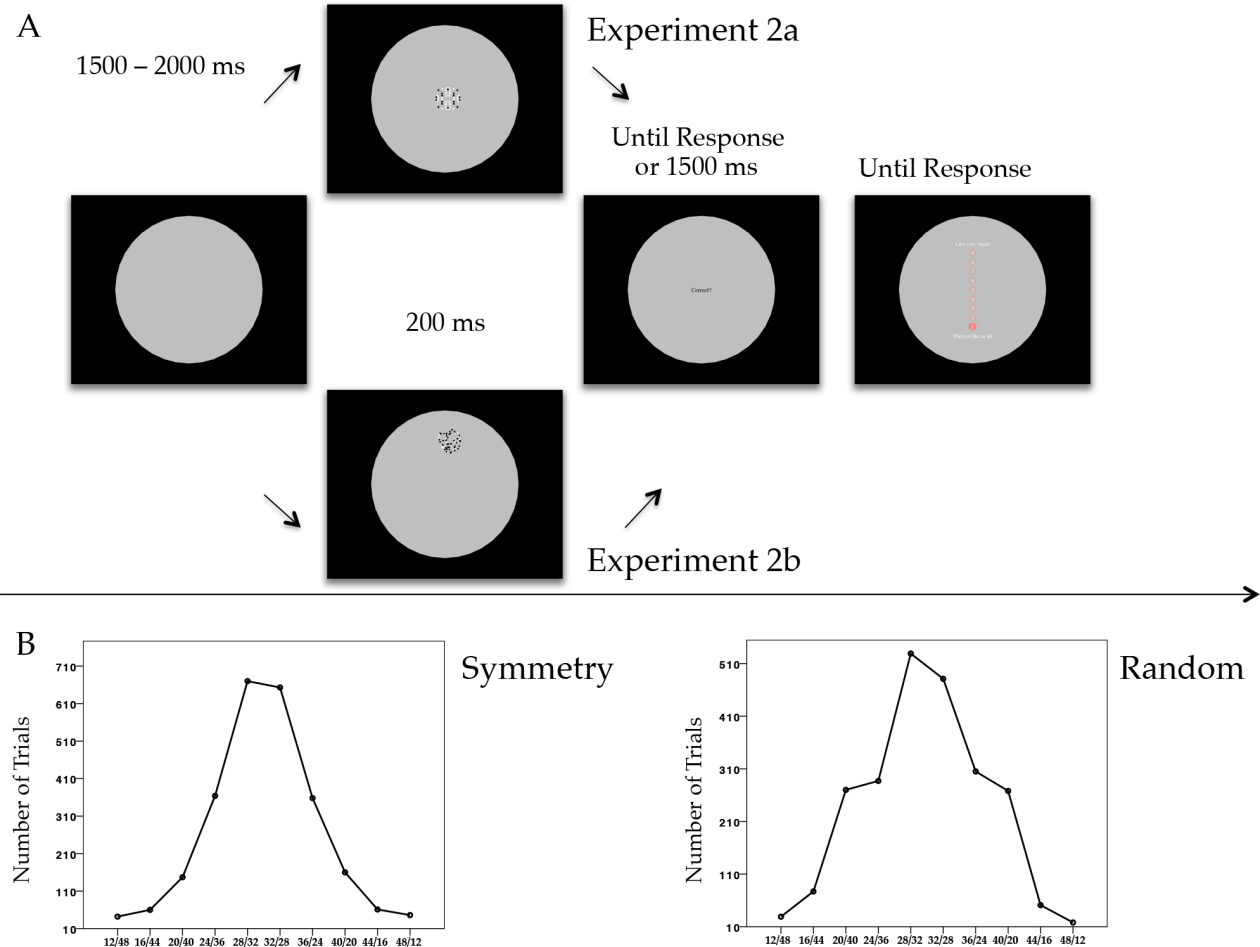
**(A)** Experimental procedure of Experiment 2a and Experiment 2b. Each trial started with an interval randomized between 1500 ms and 2000 ms. During this interval the participant could look at any location within the central large circle, and chose a point where maintaining the gaze. After the interval an abstract pattern appeared at a random location within the circle. Experiment 1a: The pattern was symmetry; Experiment 1b: The pattern was random. Patterns remained on the screen for 200 ms. Participants were encouraged to control the instinct of attempting to look at the pattern and maintain fixation on the point they chose. Immediately after offset, participants reported whether the pattern contained more black or more white dots. If no response was given within 1500 ms from pattern offset, the trial was considered null. Immediately after pattern offset, a 9-points rating scale was presented in the centre of the screen. Participants moved the cursor to select a value (1 = do not like at all; 9 = like very much). **(B)** Line graphs showing the number of trials in which different proportions of black/white dots were presented within the patterns. The program randomly generated black/white dots proportions. Ten possible configurations were generated and their frequency of appearance assumed a bell-shaped configuration. Left plot shows the black/white proportions frequency for symmetry patterns (Experiment 2a). Right plot shows the black/white proportions frequency for random patterns (Experiment 2b).

The design was similar to Experiment 1, although both stimuli and procedure presented some important differences (See Fig 4). Patterns were made by black and white dots. The proportion of black and white dots was randomly generated on each trial and was never 50–50%. In Experiment 2a all patterns were *two-fold bilateral symmetry*. In the Method session of Experiment 1, we mentioned symmetric patterns were constructed by mirroring the structure of one quadrant of the pattern in the other three quadrants. In the same way, the number of black and white dots was randomized only within one quadrant. Therefore there were limited possible configurations, based on black/white proportions. Ten different types of black/white configurations were generated (12/48, 16/44, 20/40, 24/36, 28/32, 32/28, 36/24, 40/20, 44/16, and 48/12). The frequency of appearance of the configurations was distributed with a bell-shaped configuration, as shown in Fig 4B.

Each trial started with a variable inter-trial interval (between 1.5 and 2 s) in which participants chose arbitrarily a fixation point. Similarly to Experiment 1 the pattern could appear at any position within the grey circle. Patterns remained on the screen for 200 ms. Participants pressed one button if the pattern contained more black dots and the other button if the patterns contained more white dots. Ten participants pressed the left button for ‘more blacks’ and the right button for ‘more whites’, whereas the others did the opposite. Participants were asked to be as fast and accurate as possible. Immediately after their response, participants evaluated the pattern aesthetically on a rating scale from 9 to 1. If no response was given after 1500 ms, the rating scale was presented and the trial considered null. In this Experiment, the starting point of the rating scale was the lowest number “1”, instead of the central (neutral) point “5” that was used in Experiment 1.

Note that if preference depends on eccentricity the lowest number would be used to rate the pattern at the farthest position and the highest number would be used for the foveal position. Therefore the scale works in a counter-directional manner compared to pattern position (rating the closest pattern would require to move to the farthest position on the rating scale). One might point out that starting form the highest number “9” would be more intuitive. However, this would risk making the task too obvious. Moreover, moving the cursor from the most positive value to more negative value, would represent a devaluation process instead of an evaluation process.

Each participant did an introductory session, in which 10 examples of the patterns were shown. This was followed by a practice session of 36 trials. In the practice session, feedbacks reporting the real number of black/white dot and correct/incorrect response were shown in order to help participant to familiarize with the patterns. Because we expected participants would find the task difficult, we encouraged them to try to be as accurate as possible without worrying too much about the quality of their performance. Participants were not told about the preference evaluation task until the beginning of the experimental session.

#### Data analysis

Similarly to Experiment 1, each eccentricity value was obtained by calculating the distance between the ocular coordinates at target onset and the coordinates of the centre of the pattern. Eccentricity ranged from a minimum of 0 to a maximum of 18 degrees of visual angle (M = 8.12; SD = 4.26; range = 18.3). The percentage of lost trials because of blinks and bad signal was 5.7%. The trials in which eyes movement were made during pattern presentation were removed (8.4%). In total 85% of original trials were included in the analysis.

We ran two multilevel linear models, for reaction time and preference formation respectively, and a binary logistic analysis for accuracy. Note that the model for RTs included only trials in which a correct response was made (84% of the trials), whereas the model for preference ratings included all trials. Random variables were participants and trial number. The fixed factors were: MIE, and Eccentricity.

In the analysis of preference ratings the factor MIIES was added. Moreover we also included the Number of black dots within the pattern, and the interaction Number of black dots X Eccentricity, to test whether any possible contribution of this factor on the evaluation.

#### Results and Discussion

Unlike Experiment 1, participants were exposed only to symmetric patterns and responded to another dimension (proportion of black/white dots within the pattern). Eccentricity did not predict the latency of correct responses (t_(17)_ = 1.5, p = .15; see Table 5).

**Table 5:** Results from analysis of reaction times.

	β	SE	t	p
**Intercept**	0.8	0.02	40.45	< 0.001
**MIE**	-0.002	0.02	-0.08	0.9
**Eccentricity**	0.003	0.002	1.5	0.15

Results from Multilevel Linear Model for analysis of reaction times as a function of visual eccentricity in Experiment 2a.

Overall participants gave incorrect responses on 16% of trials. We ran a Binary Logistic model for the analysis of accuracy, which showed that eccentricity did not predict lower odd ratio of correct responses (OR = .999; *Χsq* (1, *N* = 20) = 0.01, *p* = 0.7; see Table 6). The proportion of black/white dots did not affect the odds of responses overall.

**Table 6:** Results from analysis of correct responses.

	β	SE	Odd Ratio	prob	*Χsq*	p
**Intercept**	1.66	0.09				
**MIE**	0.08	0.1	1.1	0.8	0.75	0.4
**Eccentricity**	-0.001	0.01	1	0.8	0.01	0.7

Results from Binary Logistic Model for analysis of correct responses as a function of visual eccentricity in Experiment 2a.

**Fig 5:**
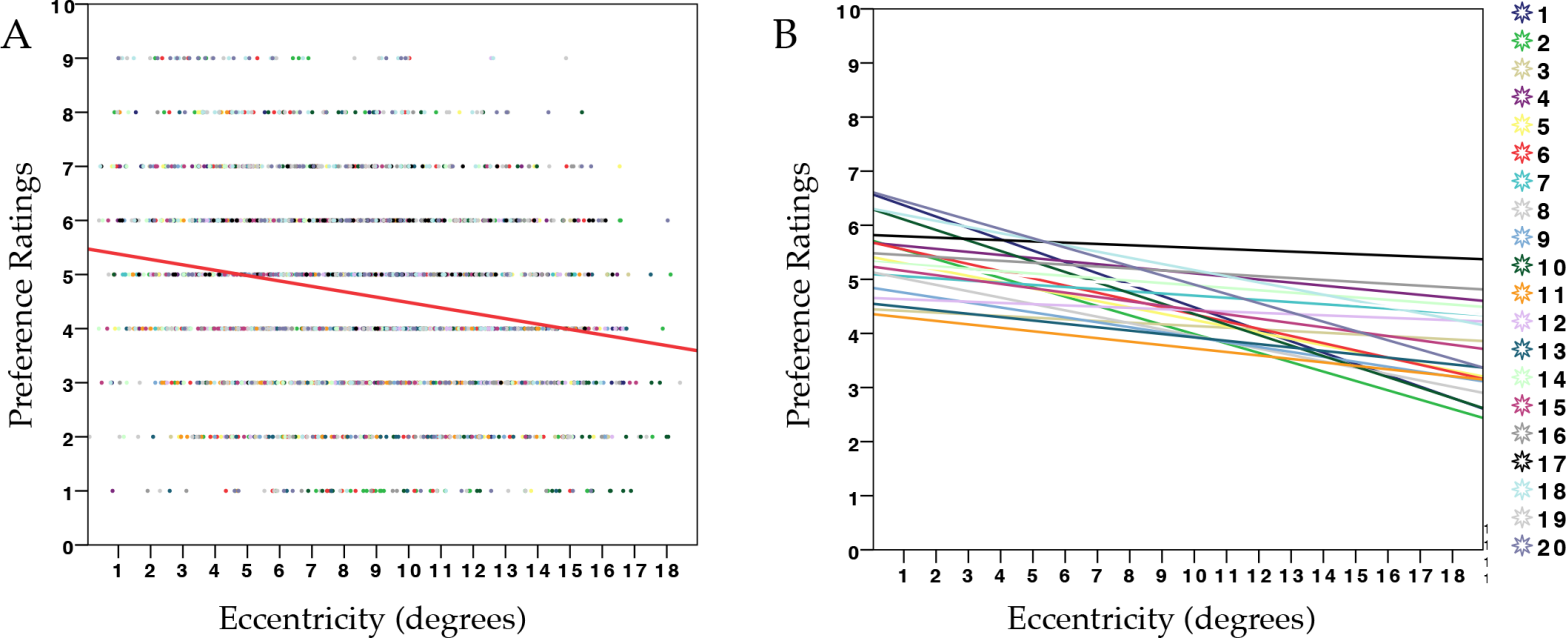
Results from Experiment 2a **(A)** Scatterplot showing preference ratings (1–9) for symmetry in relation to retinal eccentricity across all trials and participants, with regression line (red) (R2 = 0.05). **(B)** Each line corresponds to the relationship between preference and eccentricity within each participant for symmetry patterns.

This experiment challenged a possible interpretation of the results observed in Experiment 1: lower ratings for symmetry could be due to increasing difficulty in discriminating between random and symmetry. The results obtained in this experiment showed that eccentricity was a good predictor for preference evaluation (t_(14)_ = - 10.33, p <0.001), even if only one type of pattern was employed (see Table 7). Fig 5A shows preference-ratings as a function of eccentricity, whereas Fig 5B illustrates the individual regression lines. There is a trend from more positive ratings to more negative ratings with increasing eccentricity. This result suggests that the distance of the symmetrical pattern from fixation affected evaluation proportionally. This is in line with the hypothesis that liking of symmetry depends on the goodness of regularity processed around the axis of symmetry.

**Table 7:** Results from analysis of preference ratings.

	β	SE	t	p
**Intercept**	4.6	0.1	44.6	<0.001
**MIE**	-0.1	0.1	-0.8	0.4
**MIIES**	<0.001	0.001	0.1	0.9
**N black dots**	0.3	0.005	5.0	<0.001
**Eccentricity**	-0.1	0.01	-10.3	<0.001
Eccentricity * N black dots	0.001	0.001	1.0	0.3

Results from Multilevel Linear Model for analysis of preference ratings as a function of visual eccentricity in Experiment 2a.

Because participants were instructed to attend to proportion of black/white dots, this factor might have influenced ratings. The number of black dots was a good predictor of preference for the pattern (t_(14)_ = 5.00, p <0.001). However, the interaction between Number of black dots and Eccentricity was not significant (t_(14)_ = 1.0, p = 0.3). A higher proportion of black dots increased patterns’ contrast against the grey background, favouring the perception of its symmetrical structure. This could explain the higher preference for patterns with more black dots.

This experiment supports the hypothesis of a relationship between eccentricity and evaluation of symmetry. However, here participants did not explicitly attend to symmetry and we observed that the proportion of black dots within the pattern significantly affected evaluation. Experiment 2b was conducted to test the evaluation of random patterns instead of symmetry by using the same design.

### Experiment 2b

Another group of twenty participants performed the same experiment with a variation: patterns were always random. Similarly to what observed in Experiment 2a, eccentricity might predict a decrease in preference for random patterns. This would suggest that eccentricity induces more negative evaluation of abstract patterns (at least when a discrimination task is required), probably as consequence of reduced confidence. On the other hand, eccentricity might not affect evaluation for this type of pattern. This result would be in line with the hypothesis that eccentricity does not always affect aesthetic appreciation of meaningless patterns. On the contrary it specifically affects the aesthetic appreciation of symmetry.

#### Method

Design and apparatus were the same of Experiment 2a. The only change was on the type of patterns. The arrangement of dots within each quadrant was unconstrained to obtain a random configuration. However the proportion of black/white dots was controlled in order to have same number of black/white dots in each quadrant. The distribution of black/white dots was similar to Experiment 2a (see Fig 4B).

#### Data Analysis

Eccentricity ranged from a minimum of 0.1 to a maximum of 19.6 degrees of visual angle (M = 8.18; SD = 3.9; range = 19.51). The percentage of lost trials because of bad signal was 10.9%. The trials in which eyes movement were made during pattern presentation were removed (7.4%). In total, 83% of original trials were included in the analysis. Two multilevel linear models (reaction time and preference formation) and a binary logistic analysis (accuracy) were conducted, same as in Experiment 2a.

#### Results and Discussion

Multilevel linear modelling on latency of correct responses did not reveal any effect of eccentricity (t_(17)_ = 0.5, p = 0.6) (Table 8).

**Table 8:** Results from analysis of reaction times.

	β	SE	t	p
**Intercept**	0.73	0.02	31.5	<0.001
**MIE**	-0.01	0.03	-0.4	0.7
**Eccentricity**	0.001	0.002	0.5	0.6

Results from Multilevel Linear Model for analysis of reaction times as a function of visual eccentricity in Experiment 2b.

The overall percentage of incorrect responses was 24% of trials. A t-test analysis revealed that this was significantly higher than in Experiment 2a (t_(19)_ = -5.615, p <0.001).

We ran a Binary Logistic model for the analysis of accuracy, which showed that eccentricity reduced the odds of correct responses significantly (OR = .95, *Χsq* (1, *N* = 20) = 18.0, *p* < .0.001) (Table 9). This result is considerably different to what observed in Experiment 2a. Participants made significantly more errors, and accuracy worsened with increasing eccentricity in Experiment 2b. Possibly, in Experiment 2a, specular pairings of dots with same luminance polarity facilitated the estimation of correct proportion of black/white dots, even when the pattern was far from fixation. On the contrary, the random distribution of the dots in the patterns of Experiment 2b made the detection of correct proportions more difficult.

**Table 9:** Results from analysis of correct responses.

	β	SE	Odd Ratio	prob	*Χsq*	p
**Intercept**	1.2	0.08				<0.001
**MIE**	0.05	0.09	1.05	0.78	0.2	0.55
**Eccentricity**	-0.05	0.01	.95	0.76	18	<0.001

Results from Binary Logistic Model for analysis of correct responses as a function of visual eccentricity in Experiment 2b.

In a recent work, Apthorp & Bell [50] asked participants to estimate the relative number of items for symmetric and random patterns, and found that symmetry was perceived as less numerous. Our result is partially in agreement with Apthorp & Bell [50] as it also shows that symmetry influences the perception of pattern content. However, it is possible that when looking at proportion of equiluminant dots within a pattern, the presence of symmetry may favour a more accurate numerical estimation (as opposed to underestimation).

**Fig 6:**
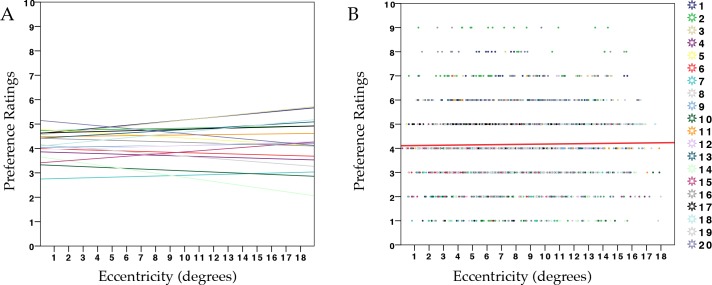
Results from Experiment 2b **(A)** Scatterdot plot showing preference ratings (1–9) for random in relation to retinal eccentricity, across all trials and participants, with regression line (red) (R2 = 0.0003). **(B)** Each line corresponds to the relationship between preference and eccentricity within each participant for random patterns.

Although increasing eccentricity significantly affected performance, the linear model on preference evaluation showed no influence of eccentricity on preference ratings (Fig 6A and 6B). In this experiment, the proportion of black/white dots did not influence evaluation. Probably eccentricity did not predict devaluation because patterns were completely random and meaningless at all eccentricities. Also different proportions of black and white dots did not affect the aesthetic appearance of the pattern at any eccentricity. Therefore eccentricity is a predictor of aesthetic appreciation but only for symmetry (even if it is always present and task-irrelevant). Table 10 shows the results.

**Table 10:** Results from analysis of preference ratings.

	β	SE	t	p
**Intercept**	4.2	0.1	29.1	<0.001
**MIE**	0.04	0.2	0.3	0.8
**MIIES**	-0.001	0.001	-1.0	0.3
**N black dots**	-0.01	0.004	-1.5	0.15
**Eccentricity**	0.005	0.01	0.7	0.5
Eccentricity * N black dots	0.001	0.001	1.0	0.3

Results from Multilevel Linear Model for analysis of preference ratings as a function of visual eccentricity in Experiment 2b.

## General Discussion

For abstract patterns, bilateral symmetry is a powerful predictor of aesthetic judgments. This is supported by a large literature, using either explicit measures [23,24] or implicit measures [26,27]. Symmetry is highly salient to the visual system, and therefore, a strong preference for symmetric configurations has been attributed to the ease of its processing (e.g. *The Perceptual Fluency Hypothesis*, [30]). However, saliency of bilateral symmetry is sensitive to several parameters. One example is retinal eccentricity. The detection of symmetry is possible at different locations in the visual field (at least when pattern regularity is the focus of the task). However, the percept of symmetry drastically reduces even with small shifts from the center of the retina [38]. Increasing eccentricity leads to a gradual decrease in performance (e.g. discriminating symmetry from non-symmetry) [38,40,51]. In this study we investigated the role of visual eccentricity on the evaluation of symmetry. Preference for bilateral symmetry might depend on the perceptual information available when symmetry is processed at fovea. Because previous studies involving preference for symmetry were conducted in central vision, preference for symmetry at different location on the retina had not been systematically investigated. This study tested preference evaluation for highly regular patterns (mirror symmetry on both vertical and horizontal axis) and highly irregular pattern (randomly arranged dots) across retinal eccentricity.

The results obtained in this study helped to answer our initial questions. Q1). Is retinal eccentricity a general predictor of lower preference or is it specifically detrimental for the aesthetic appreciation of regular patterns (bilateral symmetry)? In two experiments (Experiment 1 and Experiment 2a) we observed that the evaluation of symmetry decreased with increasing eccentricity. On the contrary, abstract random patterns were similarly evaluated at all eccentricities (Experiment 1 and Experiment 2b) (Q2). Does eccentricity affect preference evaluation through the impairment of symmetry discrimination at peripheral locations? We did not obtain evidence of an effect of eccentricity on symmetry discrimination (Experiment 1), which was measured by response time and accuracy. Moreover in Experiment 2a, symmetry was task irrelevant. Therefore, the effect of eccentricity on evaluation cannot be explained by mere difficulty in processing pattern regularity in the periphery.

As previously mentioned it has been found that shifts of 1°-2° from the fovea cause a drastic drop in sensitivity to bilateral symmetry [12,36,39,40]. As the size of the patterns was maintained unvaried, we expected to observe a worsening in performance with increasing eccentricity. The experimental design probably played a critical role in cancelling any effect of eccentricity on performance. This approach is substantially different from other studies on the saliency of symmetry in the periphery, and a number of factors might affect the way in which participants distributed attention within the circle. Stimulus locations were not decided a priori; the fixation location changed at every trial; the participant chose the location to fixate arbitrarily; there was no fixation stimulus (e.g. a cross or a point) on which to focus attention. By randomizing the point of fixation as well as pattern location within the circle, participants could not make any prediction about the location of the incoming pattern. Further investigation may reveal that integrating any of these factors in the design can lead to a gradient of response speed as function of retinal eccentricity.

Although the design employed in this study cancelled any effect of eccentricity on performance, preference evaluation was sensitive to the retinal location of the (symmetrical) patterns. This is interesting. It suggests that visual eccentricity probably affects the perceptual processing of the pattern, even though this reduced saliency cannot be reported behaviourally.

The aesthetic appreciation of symmetry therefore is a function of the degree of regularity perceived around the axis. The gradual reduction of sensitivity caused by eccentricity was reflected in more negative evaluation of symmetry.

Experiment 2 showed that this happened even when symmetry was task-irrelevant. Although participants attended to the proportion of black/white dots within the pattern, symmetry was gradually disliked across the further peripheral location. The goodness of processed symmetry was the critical factor affecting appreciation. On the contrary, irregular patterns (i.e. random) are perceptually meaningless at any distance from the point of highest visual acuity. For this reason, the task-relevant factor (i.e. proportion of dots with same luminance polarity) was the only predictor of preference modulation in Experiment 2b.

This study shows that the link between symmetry and beauty is sensitive to its location in the visual field. Although symmetry discrimination happens at any level in the periphery, symmetry appreciation is restricted to proximity to the fovea. This may explain why beauty is detected in the periphery, however it requires foveal observation to be appreciated and elicit an emotional response.
